# The influence of invariant solutions on the transient behaviour of an air bubble in a Hele-Shaw channel

**DOI:** 10.1098/rspa.2019.0434

**Published:** 2019-12-18

**Authors:** Jack S. Keeler, Alice B. Thompson, Grégoire Lemoult, Anne Juel, Andrew L. Hazel

**Affiliations:** 1 School of Mathematics and Manchester Centre for Nonlinear Dynamics (MCND), University of Manchester, Oxford Road, Manchester M13 9PL, UK; 2 School of Physics and Astronomy and MCND, University of Manchester, Oxford Road, Manchester M13 9PL, UK; 3 CNRS, UMR 6294, Laboratoire Onde et Milieux Complexes (LOMC) 53, Normandie Université, UniHavre, rue de Prony, Le Havre Cedex 76058, France

**Keywords:** Hele-Shaw channel, bifurcation analysis, weakly nonlinear analysis, edge states

## Abstract

We hypothesize that dynamical systems concepts used to study the transition to turbulence in shear flows are applicable to other transition phenomena in fluid mechanics. In this paper, we consider a finite air bubble that propagates within a Hele-Shaw channel containing a depth-perturbation. Recent experiments revealed that the bubble shape becomes more complex, quantified by an increasing number of transient bubble tips, with increasing flow rate. Eventually, the bubble changes topology, breaking into multiple distinct entities with non-trivial dynamics. We demonstrate that qualitatively similar behaviour to the experiments is exhibited by a previously established, depth-averaged mathematical model and arises from the model’s intricate solution structure. For the bubble volumes studied, a stable asymmetric bubble exists for all flow rates of interest, while a second stable solution branch develops above a critical flow rate and transitions between symmetric and asymmetric shapes. The region of bistability is bounded by two Hopf bifurcations on the second branch. By developing a method for a numerical weakly nonlinear stability analysis we show that unstable periodic orbits (UPOs) emanate from the first Hopf bifurcation. Moreover, as has been found in shear flows, the UPOs are edge states that influence the transient behaviour of the system.

## 1. Introduction

A Hele-Shaw channel consists of two parallel glass plates, separated by a distance much smaller than the width of the channel. If a trapped viscous fluid is extracted at a constant flux from one end of the channel and an air bubble is placed at the other end, then the bubble will propagate and change shape as it does so. For sufficiently large bubbles, the only stable solution is for the bubble to propagate symmetrically along the centreline of the channel, analogous to the symmetric semi-infinite air finger that develops when one end of the channel is left open to the atmosphere [1]. For higher flow rates in large aspect ratio channels, propagating air fingers develop complex patterns via multiple tip splitting events as well as side-branching [2,3]. The onset of this complex interfacial dynamics appears to be a subcritical transition, a feature that it shares with other systems including the buckling of elastica and the transition to turbulence in shear flows. Specifically, the steady symmetrically propagating solution is linearly stable for all values of the flow rate at which it has been computed, meaning that finite perturbations are required to initiate the complex dynamics. Moreover, the value of the critical dimensionless flow rate for the onset of patterns cannot be precisely determined and is very sensitive to the level of perturbation in the system: the transition occurs at lower dimensionless flow rates as the roughness of the channel walls is increased [2].

**Figure 1.**
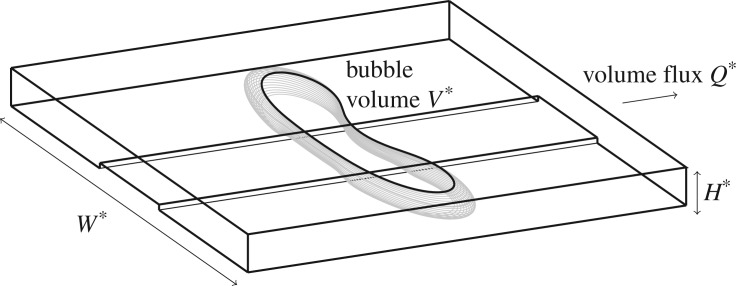
Sketch of a bubble propagating in a Hele-Shaw channel with a depth-perturbation on the bottom, showing the 3D location of the interface, depth-perturbation and direction of propagation and the dimensional quantities, W*, H*, V* and Q*.

**Figure 2.**
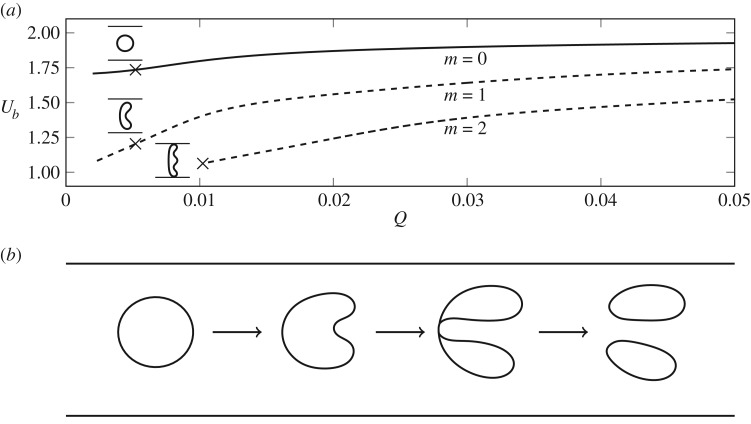
(*a*) The steady solution space for a bubble of fixed volume in an unperturbed channel with side walls and aspect ratio W*/H* = 40 showing the first three solution branches (labelled *m* = 0, 1, 2). Stable branches are solid and unstable branches are dashed. The solution measure *U*_*b*_ is the speed of bubble relative to fluid ahead, while *Q* is the dimensionless imposed flow rate. The insets indicate the bubble shapes (as shown from above) denoted by crosses on the solution branches and are on a 1:1 scale with *x*, *y* ∈ [ − 1, 1]. These solutions were calculated using the set of equations described in §2. (*b*) A sketch of an initial bubble evolving in time to form two tips and eventually break-up, yielding two separate bubbles.

In this paper, we concentrate on bubble propagation in a geometrically perturbed Hele-Shaw cell: a rectangular prism is added to the base of the channel, as sketched in figure 1. By studying bubbles, rather than air fingers, and working in a co-moving frame, we can follow long-time evolution of the system in short computational domains. This relatively simple system still exhibits a wide variety of nonlinear dynamical phenomena [4–11] and the recent experimental results of Franco-Gómez *et al.* [11] have shown that the system contains regions of bistability in which finite perturbations can provoke a range of possibilities for the time-evolution of an initially centred bubble. For small volume fluxes, the bubble will eventually reach a stable asymmetric state on one side (or the other) of the depth perturbation; but if the volume flux exceeds a critical threshold the bubble shape becomes increasingly deformed before eventually breaking up into two or more distinct parts, as sketched in figure 2*b*, despite theoretical predictions that a stable steady state exists for all flow rates, a feature preserved from the unperturbed Hele-Shaw channel. Thus, this model system allows us to explore whether the dynamical systems concepts applied in the study of transition to turbulence in shear flows also apply to the transient behaviour of other canonical problems in fluid mechanics.

We study the behaviour theoretically by finding invariant solutions, namely steady states and periodic orbits, of the system. We pursue the idea, hypothesized by Franco-Gómez *et al.* [11], that when the flux is large enough the complex time-dependent behaviour of a single bubble can be interpreted as a transient exploration of the stable manifolds of weakly unstable *edge states*. We consider only the dynamics before the changes in topology when the bubble breaks up into two or more separate bubbles: systems with their own dynamics that we do not pursue here.

In the context of fluid mechanics, an edge state is an invariant solution of the governing equations that is ‘weakly’ unstable having only a small number of unstable eigenvalues and whose, attracting, stable manifold forms the ‘boundary’ between two qualitatively different dynamical outcomes. Edge-tracking techniques have been used in a number of different scenarios including shear flow and pipe flow [12–15] and more recently droplet break-up [16]. In these studies, the edge state was found by direct numerical simulation of the governing equations and interval bisection of the initial conditions. This methodology is easy to implement numerically but can be computationally expensive and does not reveal whether the edge state corresponds to a steady state, periodic orbit or other invariant solution.

One advantage of the Hele-Shaw system is that the only nonlinearities in the system arise due to boundary conditions on the bubble because the reduced Reynolds number is extremely small. Thus the shape of the interface gives an immediate visual representation of nonlinear behaviour. Another advantage is that by assuming that the width of the channel is large compared with its height, the behaviour of the system can be described by a depth-averaged set of equations that is more amenable to analysis than the full Navier–Stokes equations [1,17]. These reduced equations (stated in §2), often called Darcy or Hele-Shaw equations, have been used in most theoretical studies of this system, and lead to predictions for the bubble shape as viewed from above (sketched in figure 2*b*). In the unperturbed system at zero surface tension, exact steady solutions can be found by conformal mapping techniques, and for each bubble volume, there is a two-parameter family of solutions described by the centroid offset and bubble speed [18–20]. The introduction of surface tension selects both a main, symmetric branch of linearly stable steady solutions that persists for all fluxes, along with a countably infinite sequence of unstable ‘exotic’ bubble shapes; these exist in channels bounded by parallel side walls [19] as well as in unbounded channels [21,22]. Numerical simulations of the time-dependent problem have also been reported [23,24]. Figure 2*a* shows the bubble shape and speed for the first three solution branches in channels with a rectangular cross section. Introducing a depth-perturbation (see sketch in figure 1) allows the solution branches to interact, which results in the diverse range of observed stable steady states and time-dependent behaviour [4–11]. In the context of air fingers, Franco-Gómez *et al.* [9] showed that this interaction occurs for small amplitudes of the perturbation (provided the aspect ratio of the channel is large) and is robust to changes in the perturbation amplitude.

We will show that the bifurcation structure of the depth-averaged equations does indeed feature bistability between steady states, and that previously unknown unstable periodic orbits (UPOs) influence whether the bubble breaks up or returns to a stable configuration. We have sufficient knowledge of the solution structure that, rather than edge tracking, we can develop a general numerical procedure for weakly nonlinear analysis that allows us to determine an approximation to the periodic orbits and steady states. We emphasize that the method described in this paper, although applied to our specific set of equations, can readily be adapted for other problems and we provide a numerical recipe for this procedure. The results of the weakly nonlinear analysis, along with time-dependent calculations, show that the previously unknown UPOs are indeed edge states of the system that influence the eventual fate of the bubble.

The paper is set out as follows. Initially, in §2, we introduce the details of the system and describe the depth-averaged governing equations, non-dimensional parameters and numerical methods. In §3, we start our investigation with a range of initial-value calculations and compare these to the previous experiments [11]. In §4, we present an extended bifurcation diagram for the invariant steady states and perform a linear stability analysis. In §5, we derive our general method for determining the weakly nonlinear approximation to the invariant periodic orbits of the system by employing the method of multiple scales near the bifurcation points. Finally, in §6, we summarize our results and briefly discuss the importance of the periodic orbits to the transient behaviour of the system and their interpretation as edge states.

## 2. Governing equations

The physical situation is shown in figure 1. The channel has dimensional outer width W* and height H*. The channel is filled with a viscous fluid of density *ρ* and dynamic viscosity *μ*, containing a single air bubble with known volume V* = (W*)^2^H*V/4, where *V* is the non-dimensional volume. Fluid flow is driven by withdrawing fluid at a constant flux Q* far ahead of the bubble. The system is non-dimensionalized based on a length scale W*/2 in the *x*- and *y*-directions, and on the velocity scale U*_0_ = Q*/(W*H*). We define the non-dimensional flow rate *Q* = μU*_0_/*γ*, where *γ* is the coefficient of surface tension at the air/fluid interface. *Q* quantifies the importance of viscosity and surface tension in this system, and so can be interpreted as a capillary number, although it does not directly relate to the bubble speed. Additionally we define the aspect ratio of the channel *α* = W*/H*. Details of the derivation of the depth-averaged equations from the Navier–Stokes equations can be found in [8,11]. Within the fluid bulk, the critical assumptions are that the channel aspect ratio *α* ≫ 1 and that the reduced Reynolds number ρU*_0_ W*/(*μα*^2^) ≪ 1.

**Figure 3.**
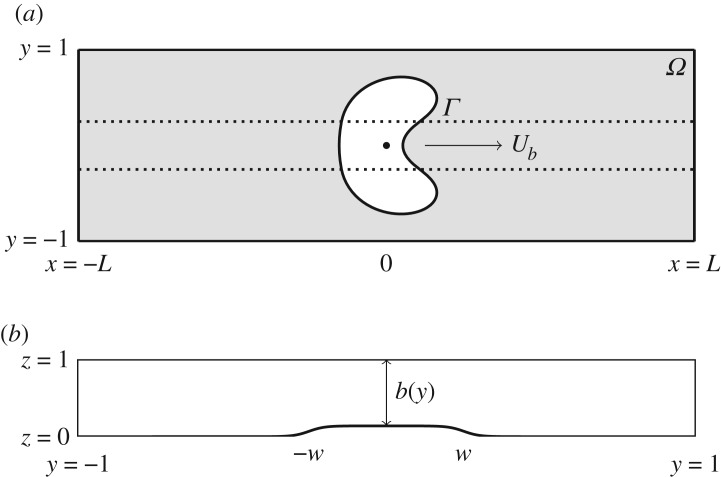
The non-dimensional computational domain. (*a*) Top-view sketch of bubble propagation, which is the focus of the depth-averaged model. Calculations take place in a frame of reference moving with the bubble centroid, and the computational domain is truncated in *x* ∈ [ − *L*, *L*] where *L* is a dimensionless distance, typically chosen to be *L* = 4 in the simulations. (*b*) For the depth-averaged calculations, a smoothed version of the depth-perturbation is used, given by (2.2), and plotted here in the (*y*, *z*) plane when *h* = 0.024, *s* = 40, *w* = 0.25.

The non-dimensional domain is shown in figure 3. The depth-averaged velocity at any point within the fluid is given by $\hat{\text{u}}=-b^{2}(y)\nabla p$, where *p* = *p*(*x*, *y*) is the fluid pressure and *b*(*y*) is the height of the channel (figure 3*b*). We work in a frame moving at dimensionless speed *U*_*b*_(*t*) chosen at each time step so that the *x* component of the bubble centroid remains fixed in this moving frame. The equations to be solved in the fluid domain and on the bubble boundary are

2.1*a* $$\begin{array}{r} \nabla \cdot (b^{3}(y)\nabla p)=0\;\text{in}\;\Omega , \end{array}$$

2.1*b* $$\begin{array}{r} p_{b}-p=\frac{1}{3}\left(\frac{1}{\alpha Qb(y)}+\frac{\kappa }{\alpha^{2}Q}\right)\;\text{on}\;\Gamma \end{array}$$

2.1*c* $$\begin{array}{rl} \;\text{and}\; & \text{n}\cdot {\text{R}}_{t}+\text{n}\cdot {\text{e}}_{x}U_{b}(t)+b^{2}(y)\text{n}\cdot \nabla p=0\;\text{on}\;\Gamma , \end{array}$$

Here, Ω denotes the two-dimensional fluid domain (the shaded region in figure 3*a*) and Γ the bubble boundary, *p*_*b*_(*t*) is the unknown pressure inside the bubble, **R** = (*x*_*b*_, *y*_*b*_) is the position of the bubble interface, *κ* is the curvature of this 2D interface, and **n** is a unit normal vector directed out of the bubble. Equation (2.1*a*) follows from mass conservation in the fluid domain. Our depth-averaged equations are second order in space, and hence on the side walls we can apply only no-penetration conditions, *p*_*y*_ = 0 on *y* = ±1. We set *p* = 0 at *x* = −*L* and the equation *p*_*x*_ = −*G* achieves the constant volume flux at *x* = *L* by imposing a constant pressure gradient that is found as part of the solution. Finally, *p*_*b*_ is chosen to ensure that the bubble volume *V* satisfies

∫b(y)R⋅n dΓ=V.

The most significant assumptions in the derivation of our depth-averaged model concern the conditions at the bubble boundary (2.1*b*) and (2.1*c*). For the kinematic condition (2.1*b*), we assume that the bubble occupies the full height of the channel. The pressure jump (2.1*c*) is based on the Young–Laplace condition, with the curvature of the 3D interface treated as the sum of the lateral curvature as viewed from above and the transverse curvature assumed to be the curvature of a semi-circle filling the full channel height. We neglect any effects of gravity under the assumption that the Bond number $\text{Bo}=\rho gH^{*2}/\gamma$ is small. Homsy [25] and Reinelt [26] presented corrections to (2.1*c*) for low capillary number. In the limit of zero capillary number, these corrections have the effect of multiplying the lateral curvature *κ* in (2.1*c*) by a factor of *π*/4. We have omitted this prefactor because we do not work at vanishingly small capillary number, and the corrections may also be altered by the presence of the obstacle. However, this prefactor could be recovered by rescaling *α* and *Q* and so would not qualitatively change our results.

Following previous papers, we model the depth-perturbation by a smoothed profile

2.2 $$b(y)=1-\frac{1}{2}h[\tanh(s(y+w))-\tanh(s(y-w))].$$

A typical example of this smoothed profile is shown in figure 3*b*. The dimensionless depth-perturbation height *h* can be viewed as a controlled, axially uniform perturbation of the rectangular channel. The topography profile *b*(*y*) enters the equations through the bulk equation (which is no longer amenable to conformal mapping), the kinematic boundary conditions and the variable transverse curvature of the interface. The unknowns of the problem are [*p*, **R**, *p*_*b*_, *U*_*b*_] and the control parameters are [*Q*, *V*, *h*, *w*, *s*, *α*]. An additional solution measure is the centroid of the bubble, denoted $\bar{y}$, which is useful in determining the symmetry of the system. (2.1) are solved by a finite-element discretization, using the open-source software oomph-lib [27]. Details of the implementation are given in [8] for the case of a propagating air-finger and [11] for an air bubble.

It is convenient to keep *h*, *w* and *α* constant and analyse the system mainly through variations in *Q* and *V*, which are easily manipulated in a physical, experimental set-up. Unless otherwise specified the results presented in this paper are compared with the values used by Franco-Gómez *et al.* [11] and hence a bubble of fixed volume *V* is chosen so that the projected area is *A* = *πr*^2^ with *r* = 0.46, together with a perturbation height *h* = 0.024, width *w* = 0.25, sharpness *s* = 10 and aspect ratio *α* = 40.

## 3. Initial-value calculations

**Figure 4.**
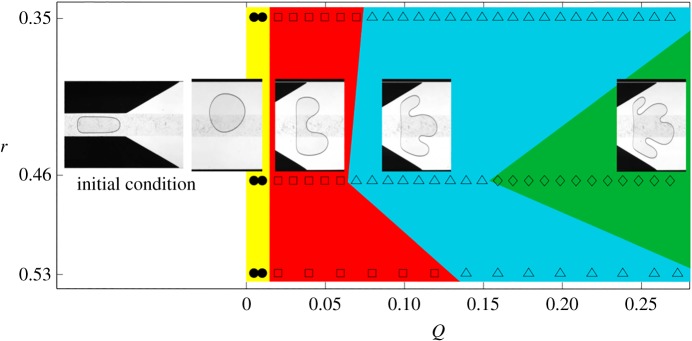
Figure adapted with permission from fig. 10 of [11], classifying the fate of circular bubbles with static diameter, 2*r* and non-dimensional flow-rate *Q* = μU*_0_/*γ* according to whether they develop one, two, three or four tips before the bubble has either broken up (hollow symbols) or remains in a steady mode of propagation (filled symbols, only at the lowest flow rate). These data are from experiments with *α* = 40, *h* = 0.024 and *w* = 0.25.^1^ (Online version in colour.)

Franco-Gómez *et al.* [11] performed a range of experiments which showed that an initially circular bubble would settle to a stable state below a critical flow rate while above this threshold the bubble becomes increasingly deformed, resulting in a variety of transient outcomes (see figure 4), including topological break-up, despite evidence to suggest that an asymmetric stable state existed for all flow rates.

**Figure 5.**
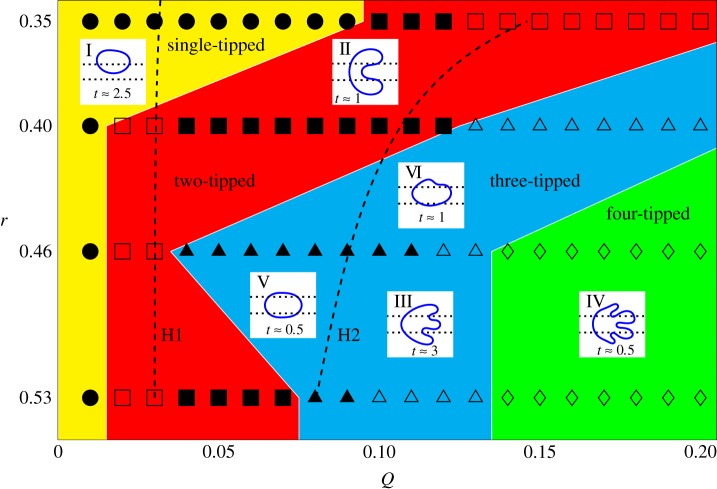
Summary of time-dependent numerical simulations starting from a circular bubble with radius (in the *xy* plane) *r* and a fixed small offset, *y*_*c*_ = 0.01. Solid symbols indicate that the bubble evolves towards either an asymmetric steady state (circles), symmetric steady state (squares) or stable oscillatory motion (triangles), labelled by i, v and vi, respectively, while hollow symbols indicate bubble break-up. In the region with inset I, the bubble always tend towards a single-tipped, steady propagation mode. In the region with inset II, for small flow rates the bubble forms two tips (ii) and breaks up (hollow symbols) or reaches a symmetric steady state (solid symbols). In the region with insets III, IV and VI, the bubble forms three tips before break-up (iii) or reaching either a symmetric steady state or a stable oscillatory state. Finally, in the region with inset IV, the initial transients involve four-tipped bubbles before breaking up (iv). The time quoted in the profiles is an indicative dimensionless time scale at which the behaviour in that particular region is typically observed. The dashed lines indicate the location of the two Hopf bifurcations in the steady solution space that are discussed in §4. (Online version in colour.)

**Figure 6.**
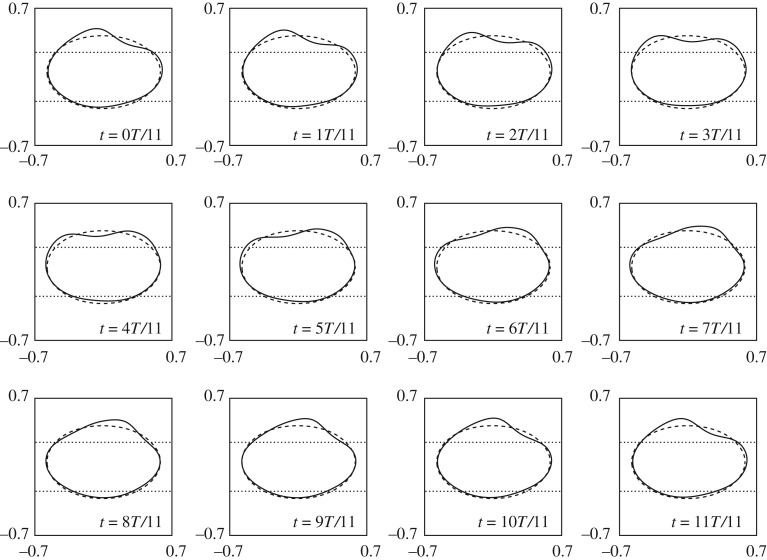
Stable periodic orbit near H2 over one period. The dashed shape is the fully nonlinear steady state solution. Bubble shapes for $Q^{-1}=Q_{2}^{-1}(1-\varepsilon^{2})$, where *Q*_2_ is the critical value for H2 and ε = 0.2. The period is given by *T* = 2*π*/*ω* where *ω* = *ω*_*c*_ + ε^2^(*c* − *ad*/*b*), which in this case is *T* = 0.5429.

In order to investigate the potential range of evolution in our model we perform a number of numerical simulations designed to mimic these experiments. The nonlinear dynamics can be examined by recording the bubble shape as it propagates along the channel. We do this by solving (2.1) with the initial condition of a circular bubble with radius *r* centred at (*x*_*c*_, *y*_*c*_) = (0.0, 0.01) and released from rest. Our simulations reveal a number of qualitatively distinct modes of propagation. The eventual propagation mode selected has a dependence on the flow rate *Q* and the radius *r* which sets the bubble volume *V*. A summary of the qualitative behaviour is shown in figure 5, with regions of parameter space classified according to the characteristic shape first adopted by the bubble. As *Q* and *V* are varied, we find that the first emergent shape may have between one and four tips. The start of each different coloured region as *Q* increases indicates the place in parameter space where the bubble first develops two, three or four tips for a given bubble volume. Single-tipped bubbles evolve smoothly to an asymmetric steadily propagating configuration (see inset labelled i in figure 5), while multiple-tipped bubbles may self-intersect in the simulations and thus break up into one or more fragments, in which case the simulations are terminated (see insets labelled ii, iii, iv in figure 5) . In some regimes, the bubble may exhibit oscillatory behaviour before decaying to either a steady state (see inset v in figure 5) or stable periodic oscillations (see inset vi for a snapshot of the oscillation and figure 6 for a weakly nonlinear approximation to a complete period of oscillation). The nature of the oscillatory modes that tend to a stable state is dependent on the flow rate in that for lower flow rates (*Q* ≈ 0.05) the bubble appears to oscillate around a symmetric steady state, while for larger flow rates (*Q* ≈ 0.1) the oscillations manifest as ‘waves’ above one edge of the depth-perturbation with the opposite side of the asymmetric bubble above the other edge of the depth-perturbation, as seen in inset vi in figure 5.

We can compare these numerical results to the experimental data of Franco-Gómez *et al.* [11] shown in figure 4. Although the boundaries between regions are quantitatively different in figures 4 and 5, the phenomenological behaviour is in agreement, and the transitions between different modes of propagation occurs over similar ranges of *Q* and *V*. For nearly all values of *Q* explored in figure 5, varying the bubble volume at fixed *Q* allows us to access a range of qualitatively different modes of propagation.

As demonstrated by these numerical time simulations, there is a large range of possible outcomes from an initially circular bubble, including oscillatory transients and multiple stable steady states. To understand this further, we now perform a detailed analysis of the steady-state bifurcation structure.

## 4. Bifurcation structure for steady states

**Figure 7.**
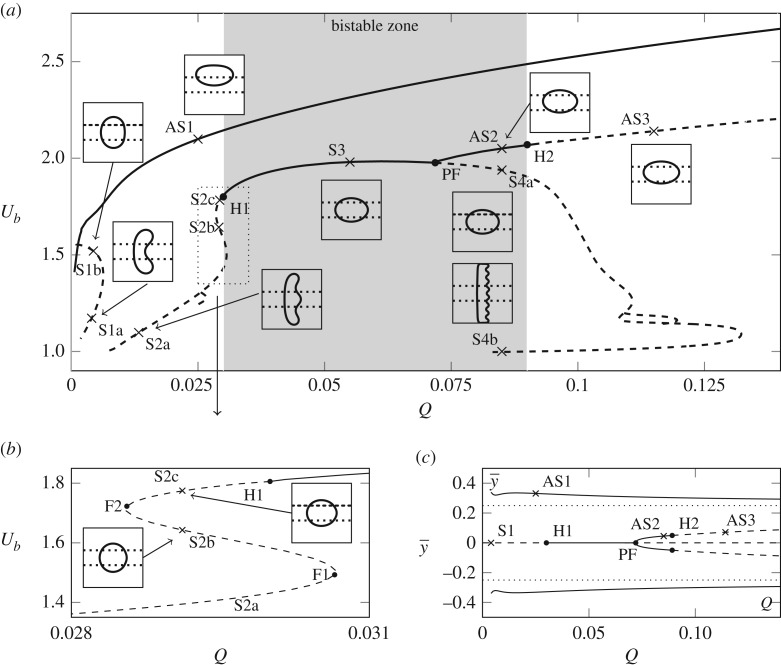
(*a*) The steady solution space in the (*Q*, *U*_*b*_) projection for a bubble of volume *V* = 0.665. The solid curves indicate stable solutions while the dashed lines indicate unstable solutions. The pitchfork and two Hopf bifurcations are denoted by solid circles and the inset diagrams correspond to points marked with a cross, plotted on a 1:1 scale in the range *x*, *y* ∈ [ − 1, 1]. S1a,b solutions *Q* = 0.004, AS1 solution *Q* = 0.025, S2a,b,c solutions, *Q* = 0.029, S3 solution *Q* = 0.055, AS2 solution *Q* = 0.085, S4a,b solutions *Q* = 0.085, AS3 solution *Q* = 0.115. The region contained in the dotted rectangle is enlarged and shown in (*b*). (*c*) The steady solution space in the $\left(Q,\bar{y}\right)$ projection.

In this section, we analyse the steady solution space. We use the flow rate, *Q*, as a continuation parameter for a fixed bubble volume and record the bubble speed, *U*_*b*_, and the centroid position, $\bar{y}$, as convenient solution measures. We also analyse the linear stability of the numerical solutions as the solution branches are traced out by solving a series of generalized eigenvalue problems numerically, see appendix A in [8]. The solution space is shown in figure 7 in the (*Q*, *U*_*b*_) and $\left(Q,\bar{y}\right)$ projections, respectively, with stable (unstable) solution branches denoted by solid (dashed) lines. We note that the branches in this steady solution space are connected through several bifurcations, in contrast to the *h* = 0 case (figure 2*a*), where the solution branches are disjoint.

For small *Q*, there are three distinct branches, as shown in figure 7. The ‘upper’ branch (with largest *U*_*b*_), AS1, is stable, characterized by an asymmetric bubble shape, and persists for all values of *Q* that were sampled. The other two branches, denoted as S1a and S1b, correspond to symmetric double-tipped solutions. As the value of *h* is decreased to zero the S1a branch approaches the *m* = 0 solution sketched in figure 2*b* while the lower part becomes the double-tipped *m* = 1 solution. The S1 branch only exists for a small range of the parameter *Q*, in contrast to the case with no depth-perturbation where the solution persists for all *Q*. Both parts of the S1 branch are unstable; the segment with larger *U*_*b*_ has one unstable eigenmode, while the slower segment is doubly unstable. Note that although it appears from figure 7*a* that the S1 and AS1 branches intersect at small *Q*, these branches are actually disjoint as can be seen by the projection of the solution onto the $\left(Q,\bar{y}\right)$ plane in figure 7*c*.

For Q⪆0.01, we are able to compute a three-tipped symmetric solution, S2a, which is unstable. As *Q* increases this branch undergoes two fold bifurcations. The first fold, F1 (shown in figure 7*b*), separates the S2a and S2b branches and the second fold, F2, separates the S2b and S2c branches. By decreasing *h* to zero, the S2a branch converges to a triple-tipped solution branch (*m* = 2 as shown in figure 2), the S2b branch becomes the double-tipped solution branch (*m* = 1) and the S2c branch becomes the stable solution branch (*m* = 2). This transition sequence is likely to involve interaction between the AS1, S1 and S2 branches. All of the S2 branches are unstable; however, for this bubble volume and channel geometry, the solution finally stabilizes via a Hopf bifurcation H1 just beyond the second fold point. The stable branch of steady solutions that results from this Hopf bifurcation is labelled S3.

As *Q* increases further on the S3 branch, a supercritical pitchfork bifurcation (PF) occurs where the S3 solution breaks symmetry and two stable asymmetric branches, denoted AS2, emanate from the bifurcation (see figure 7). Beyond the pitchfork, the unstable symmetric branch experiences multiple bifurcations as *Q* is varied and becomes increasingly unstable. This collection of unstable branches is denoted by S4. The most extreme shapes on the S4 branch, with *U*_*b*_ close to 1, correspond to the eight-tipped solution, as seen in figure 7. Note the loops on the S2 branch around *Q* ≈ 0.11 where an asymmetric branch connects to the main S4 branch via two pitchfork bifurcations. This is reminiscent of the so-called snakes and ladders bifurcations seen in other nonlinear systems; see, for example, [28,29]. This branch has at least two unstable eigenmodes and as we do not believe it affects the transient behaviour of the system we do not pursue the details of this branch here. For the largest *Q* in our calculations (*Q* = 0.15), two distinct asymmetric states persist in the (*Q*, *U*_*b*_) projection, (four in the $(Q,\bar{y}$) projection). The AS1 branch is always stable but the stable AS2 branch experiences a Hopf bifurcation, denoted by H2, and becomes unstable, the resulting branch labelled AS3.

In contrast to the *h* = 0 solution space, where only one solution is ever stable, in our case there is a finite-width region of bistability (with respect to steady states) between the H1 and H2 bifurcations. The AS1 solution is always stable but the nature of the second stable solution changes from symmetric (the S2 branch) to asymmetric (the AS2 branch), via the pitchfork bifurcation. The symmetric stable solution (S2) has been observed before in this system, see Franco-Gómez *et al.* [11], but its transition to an asymmetric stable solution as *Q* increases, and indeed the presence of the two Hopf bifurcations, is a new observation.

**Figure 8.**
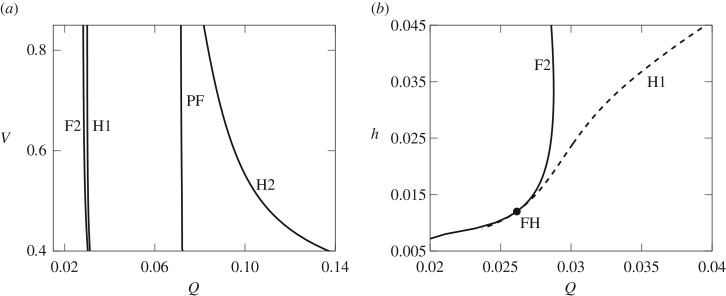
(*a*) The location of the bifurcations in the (*Q*, *V*) projection with fixed *h* = 0.024. F2 indicates the fold bifurcation that occurs before the Hopf bifurcation H1 and PF indicates the pitchfork bifurcation just before H2. (*b*) The location of the bifurcations in the (*Q*, *h*) projection with *V* fixed at *V* = 0.665. The FH point indicates a fold-Hopf bifurcation.

The results presented here are for a fixed volume, *V*, and fixed depth-perturbation height, *h*, but we can use bifurcation tracking calculations to investigate the robustness of the location and order of the bifurcations F2, H1, PF and H2 that bound the bistable region. The locations of these bifurcations as functions of *V* and *h* are shown in figure 8. We find that the ordering of the F2, H1, PF and H2 bifurcations are very robust to changes in volume and, with the exception of H2, their position is fixed (figure 8*a*). As *V* decreases, H2 migrates to larger values of *Q* and hence the width of the bistable region increases. All the bifurcations are more sensitive to *h* than *V*, and when *h* < *h*_*c*_ ≈ 0.012, the change in stability occurs at the fold, F2, instead of the Hopf, H1 (figure 8*b*). A fold-Hopf bifurcation occurs when *h* = *h*_*c*_. The dynamics of the system near this type of co-dimension two bifurcation are very complex and can lead to the appearance of invariant tori and heteroclinic orbits; see, for example, [30].

The time-dependent behaviour explored in §3 can now be interpreted in terms of the bifurcation structure. For all flow rates, there is an asymmetric stable mode of propagation, which corresponds to the AS1 branch. The region of bistability appears to coincide with the solid symbols in figure 5, which denote that the bubble is evolving towards a steady solution. The multi-tipped modes of propagation may relate to the presence of the unstable S1, S2 and S4 branches, which the time-dependent solution explores transiently, while the oscillating modes of propagation may be expected to arise due to the Hopf bifurcations. However, the linear stability analysis does not reveal the centre, nor the stability and size of the periodic orbits emanating from the Hopf bifurcations and thus the criticality of H1 and H2 remains unknown.

There are at least three possible approaches to determine the periodic orbits. If the periodic orbit is stable, we expect that for suitable initial conditions, the periodic orbit is attracting and hence initial-value simulations will eventually converge towards the periodic orbit. These simulations may be expensive if the convergence rate is slow, and in any case will not capture UPOs. The second approach is to calculate the periodic orbits directly by solving an extended system of equations as proposed by, for example, [31]. This direct solution will capture UPOs, but leads to a significantly larger system of equations and requires a discretization in both space and time. The third approach is to perform a numerical weakly nonlinear stability analysis near the Hopf points, leading to an analytic normal form of the perturbation equations near the Hopf points with numerically computed coefficients, providing an approximate expression for the periodic orbits in terms of eigenfunctions. This latter approach is the least computationally expensive and has the advantage of providing semi-analytic approximations for the amplitude, period and location of the periodic orbits and we pursue this analysis in the next section.

## 5. Weakly nonlinear stability

The aim of this section is to perform a local analysis near the Hopf bifurcation points to obtain approximations for the location, stability and size of the periodic orbits. In linear stability theory, the growth of the perturbation occurs on a single time scale and is determined by the eigenvalues alone and not the amplitude of the perturbation. In weakly nonlinear stability theory near a marginally stable solution (i.e. bifurcation point) the growth/decay of a perturbation is assumed to happen over two time scales, the eigenvalue providing the growth/decay on the fast scale *t*, while the amplitude varies on a slower time scale. The main objective of a weakly nonlinear analysis is to obtain an evolution equation for this unknown amplitude function. This method has been applied to a number of specific examples in physics, including shear flows, shallow water waves, thermoacoustics and magnetohydrodynamics; see, for example [32–38]. In these examples, the nature of the equations analysed means that the algebra often becomes very complicated. Our approach (inspired by [35] who derived a weakly nonlinear model for a different type of bifurcation) is to perform the analysis for a general set of equations that can be applied to a wide range of systems so that an analytic approximation for the periodic orbits and steady states can be obtained. The analysis is based on a continuous set of equations that are independent of the nature of a discretization procedure. We then describe a versatile numerical procedure that can be implemented to obtain semi-analytic expressions for the periodic orbits and steady states. We examine a set of equations of the form

5.1 $$R(\dot{u},u,\beta )=0,$$

where R is a nonlinear function that depends on the state variables u∈U, where U is an appropriately defined Hilbert space, time derivatives $\dot{u}$ and a parameter β∈R. The state variable, *u*, will in general depend continuously on spatial coordinates *x* and temporal coordinates *t*. To proceed further, it is assumed that the set of equations can be separated so that

5.2 $$R(\dot{u},u,\beta )\equiv M[u]\dot{u}+\beta F[u]+G[u]=0,$$

where M is a linear mass operator and F, G are nonlinear operators on the state variables independent of the parameter *β*. The form of the equations in (5.2), while not completely general, is representative of a large number of physical systems where time derivatives appear in linear combinations, including the equations that form the subject of this paper, (2.1).

We are interested in the nonlinear evolution of a perturbation near a Hopf point. A standard linearization procedure about a steady solution, *u*_*s*_, will yield a generalized eigenproblem that can be solved to find the eigenmodes, denoted *g*, and corresponding eigenvalues, denoted *s*. A Hopf point occurs at a particular value of the parameter, *β*_*c*_ say, such that a single pair of eigenvalues are located on the imaginary axis, i.e. of the form *s* = ±i*ω*_*c*_. Near this point of marginal stability the oscillations will occur on a fast time scale and will be modulated by a time-dependent amplitude function that operates on a slower time scale. Following [34,35,39] we define a fast time scale, *t*_0_ = *t*, and a slow time scale, *t*_1_ = ε^2^ *t*, where ε ≪ 1 is a small unfolding parameter, and employ the method of multiple scales. The solution, *u*, is now a function of two different time scales as well as the spatial variables denoted by *x*. The solution, time derivative and parameter *β* are therefore expanded in ascending powers of ε:

5.3*a* $$\begin{array}{rl} & u=u_{s}(x)+\varepsilon u_{1}(t_{0},t_{1},x)+\varepsilon^{2}u_{2}(t_{0},t_{1},x)+\varepsilon^{3}u_{3}(t_{0},t_{1},x)+O(\varepsilon^{4}), \end{array}$$

5.3*b* $$\begin{array}{r} \frac{\partial }{\partial t}=\frac{\partial }{\partial t_{0}}+\varepsilon^{2}\frac{\partial }{\partial t_{1}}+O(\varepsilon^{4}) \end{array}$$

5.3*c* $$\begin{array}{rl} \;\text{and}\; & \beta =\beta_{c}(1+\delta \varepsilon^{2}). \end{array}$$

The parameter *δ* = ±1 signifies which ‘side’ of the Hopf bifurcation is being analysed. Substituting the expressions in (5.3) into (5.2) yields a sequence of linear problems that can be solved at each order of ε.

Examining terms of $O(\varepsilon^{0})$ in the expansion gives the following equation to solve for the steady base solution,

5.4 $$\beta_{c}F[u_{s}]+G[u_{s}]=0.$$

With *u*_*s*_ known, we continue further and at O(ε) we have

5.5 $$L\left(\frac{\partial u_{1}}{\partial t_{0}},u_{1}\right)\equiv M[u_{s}]\frac{\partial u_{1}}{\partial t_{0}}+J[u_{s}]u_{1}=0,$$

where the operator $J[u_{s}]$ is the Jacobian operator defined by its action on $v_{1}\in U$ by the Fréchet derivative,

5.6 $$J[u_{s}]v_{1}\equiv D_{v_{1}}K[u_{s}]\equiv \lim_{k\to 0}\frac{K[u_{s}+kv_{1}]-K[u_{s}]}{k},$$

where $K[u]\equiv \beta_{c}F[u]+G[u]$. We separate *u*_1_ into spatial and temporal parts so that $u_{1}(t_{0},t_{1},x)=A(t_{1})g(x){\text{e}}^{st_{0}}$ and equation (5.5) becomes a generalized eigenproblem. Its solution determines the linear stability of the steady solutions solved in (5.4). There are an infinite number of solutions to (5.5) but as we are only interested in periodic solutions arising from a Hopf bifurcation, we choose *u*_1_ to be

5.7 $$u_{1}=A(t_{1})\;g(x)\;{\text{e}}^{\text{i}\omega_{c}t_{0}}+\text{c. c.},$$

where *A*(*t*_1_) is the undetermined amplitude of the perturbation depending on the slow time scale *t*_1_. The function *g*(*x*) is a complex eigenfunction of the generalized eigenvalue problem, $\text{i}\omega_{c}Mg=Jg$. Proceeding to $O(\varepsilon^{2})$, the problem to be solved is

5.8 $$L\left(\frac{\partial u_{2}}{\partial t_{0}},u_{2}\right)=-M[u_{1}]\frac{\partial u_{1}}{\partial t_{0}}-\delta \beta_{c}F[u_{s}]-\frac{1}{2}H[u_{s}](u_{1},u_{1}),$$

where $H[u_{s}]$ represents the bilinear Hessian operator defining its action on $v_{1},v_{2}\in U$ and using (5.6) by

5.9 $$H[u_{s}](v_{1},v_{2})\equiv D_{v_{2}}(J[u_{s}]v_{1})\equiv \lim_{k\to 0}\frac{J[u_{s}+kv_{2}]v_{1}-J[u_{s}]v_{1}}{k}.$$

The terms on the right-hand side of (5.6) are either formed of products of *u*_1_ or have no time dependence. It can be shown that the products of *u*_1_, defined in (5.8), introduce time-dependent terms proportional to $\text{exp}(2\text{i}\omega_{c}t_{0})$ in (5.7) and these do not resonate with the homogeneous solutions of the operator L. By examining the form of *u*_1_ in (5.8), a particular solution to (5.8) is sought in the form

5.10 $$u_{2}=A^{2}\varphi_{0}\;{\text{e}}^{2\text{i}\omega_{c}t_{0}}+\text{c. c.}+|A{|}^{2}\varphi_{1}+\varphi_{2},$$

where the functions φ_*i*_ are to be determined. Substituting (5.10) into (5.8) and then equating coefficients of *A*^2^, |*A*|^2^ and constant terms leads to three linear equations that can be solved to find the undetermined functions φ_*i*_ in (5.10). These equations can be stated as

5.11*a* $$\begin{array}{rl} & (2\text{i}\omega_{c}M[u_{s}]+J[u_{s}])\varphi_{0}=-\text{i}\omega_{c}M[g]g-\frac{1}{2}H[u_{s}](g,g), \end{array}$$

5.11*b* $$\begin{array}{rl} & J[u_{s}]\varphi_{1}=-\frac{1}{2}H[u_{s}](g,g^{*})-\frac{1}{2}H[u_{s}](g^{*},g)-\text{i}\omega_{c}M[g^{*}]g+\text{i}\omega_{c}M[g]g^{*} \end{array}$$

5.11*c* $$\begin{array}{rl} \;\text{and}\; & J[u_{s}]\varphi_{2}=-\delta \beta_{c}F[u_{s}], \end{array}$$

where the star denotes complex conjugation. The amplitude function *A*(*t*_1_) remains undetermined at this order. Therefore, the analysis continues to the next order, $O(\varepsilon^{3})$. The corresponding equation to be solved is

5.12 $$\begin{array}{rl} L\left(\frac{\partial u_{3}}{\partial t_{0}},u_{3}\right) & =-M[u_{2}]\frac{\partial u_{1}}{\partial t_{0}}-M[u_{1}]\frac{\partial u_{2}}{\partial t_{0}}-M[u_{s}]\frac{\partial u_{1}}{\partial t_{1}}\\ & \;-\delta \beta_{c}J_{F}[u_{s}]u_{1}-\frac{1}{2}H[u_{s}](u_{1},u_{2})-\frac{1}{2}H[u_{s}](u_{2},u_{1})-\frac{1}{6}T[u_{s}](u_{1},u_{1},u_{1}), \end{array}$$

where $J_{F}[u_{s}]u_{1}\equiv D_{u_{1}}F[u_{s}]$ and the operator T[u] is the trilinear third-order differential operator defined by its action on $v_{1},v_{2},v_{3}\in U$ and using (5.9) by

5.13 $$T[u](v_{1},v_{2},v_{3})\equiv D_{v_{3}}(H[u_{s}](v_{1},v_{2}))\equiv \lim_{k\to 0}\frac{H[u_{s}+kv_{3}](v_{1},v_{2})-H[u_{s}](v_{1},v_{2})}{k}.$$

At this order, the products formed on the right-hand side of (5.12) will result in time-dependent terms proportional to $\text{exp}(\text{i}\omega_{c}t_{0})$ and these *are* resonant with homogeneous solutions of the operator L. Therefore, a solvability condition is invoked. In linear operator theory, by the Fredholm alternative (see, for example, [40]), a linear system of the form Lu=f will either have (a) a unique solution *u*, or (b) a non-trivial solution to $L^{†}v=0$. For condition (a) to hold $\langle v^{†},f\rangle =0$. The dagger superscripts indicate the adjoint problem. The adjoint problem and inner product on the Hilbert space, *U*, are defined by

$$\langle Lu,v\rangle =\langle u,L^{†}v\rangle \;\text{and}\;\langle u,v\rangle =\int_{0}^{T}\int_{x\in \Omega }uv^{*}\;\text{d}x\;\text{d}t_{0}$$

for any functions *u* and *v* belonging to the solution function space, where Ω is the domain of definition of the spatial variable *x*, and *T* is the period of oscillation in the fast time scale *t*_0_. Applying the Fredholm alternative to (5.12) yields a solvability condition which provides a constraint on the amplitude function *A*(*t*_1_). This can be written as

5.14 $$\hat{\nu }\frac{\partial A}{\partial t_{1}}+\hat{\lambda }A+\hat{\mu }A|A{|}^{2}=0,$$

which is the weakly nonlinear Landau equation. The coefficients, $\hat{\nu }$, $\hat{\lambda }$ and $\hat{\mu }$ are defined by

5.15 $$\hat{\nu }=\langle M[u_{s}]g,g^{†}\rangle ,\;\hat{\lambda }=\sum_{k=0}^{3}\langle \Gamma_{k},g^{†}\rangle \;\text{and}\;\hat{\mu }=\sum_{k=0}^{7}\langle \Lambda_{k},g^{†}\rangle ,$$

where $g^{†}$ is the eigenfunction corresponding to the adjoint problem, $L^{†}g^{†}=0$. The values of Λ_*k*_ and Γ_*k*_ are defined as

5.16 $$\begin{array}{rl} & \Gamma_{0}=\delta \beta_{c}J_{F}[u_{s}]g,\;\Gamma_{1}=\text{i}\omega_{c}M[\varphi_{2}]g,\;\Gamma_{2}=\frac{1}{2}H[u_{s}](g,\varphi_{2}),\;\Gamma_{3}=\frac{1}{2}H[u_{s}](\varphi_{2},g),\\ & \Lambda_{0}=-\text{i}\omega_{c}M[\varphi_{0}]g^{*},\;\Lambda_{1}=\text{i}\omega_{c}M[\varphi_{1}]g,\;\Lambda_{2}=2\text{i}\omega_{c}M[g^{*}]\varphi_{0},\;\Lambda_{3}=\frac{1}{2}H[u_{s}](g,\varphi_{1}),\\ & \Lambda_{4}=\frac{1}{2}H[u_{s}](g^{*},\varphi_{0}),\;\Lambda_{5}=\frac{1}{2}H[u_{s}](\varphi_{1},g),\;\Lambda_{6}=\frac{1}{2}H[u_{s}](\varphi_{0},g^{*})\\ \;\text{and}\; & \Lambda_{7}=\frac{1}{6}T[u_{s}](g^{*},g,g)+\frac{1}{6}T[u_{s}](g,g^{*},g)+\frac{1}{6}T[u_{s}](g,g,g^{*}). \end{array}\}$$

The values of $\hat{\nu },\hat{\lambda },\hat{\mu }$ can be obtained once the functions φ_*i*_ and the eigenmodes *g* and $g^{†}$ have been calculated. For convenience, the eigenmodes *g* and $g^{†}$ are normalized so that $|\langle Mg,g^{†}\rangle |=1$. Therefore, |*ν*| = 1. Furthermore, it is more convenient to write the Landau equation as

5.17 $$\frac{\partial A}{\partial t_{1}}=\lambda A+\mu A|A{|}^{2},$$

where $\lambda =-\hat{\lambda }/\hat{\nu }$ and $\mu =-\hat{\mu }/\hat{\nu }$.

The analysis up to this point has been based on a spatially and temporally continuous set of equations. We now describe a procedure, independent of the choice of discretization, appropriate for a spatially discretized version of the equations in (5.1). In the description of the algorithm that follows, **J** and **M** are the matrix representations of J and M, respectively, and bold symbols are the discretized vectors of their continuous counterpart. It is assumed that efficient numerical linear solvers (for example, SuperLU [41]), generalized eigensolvers (for example, Trilinos [42]) and continuation algorithms are available. We note that for high-dimensional systems, these calculations are computationally demanding, but they need to be performed only once for each bifurcation point. The procedure is as follows:

(i) Find a Hopf bifurcation as *β* is varied by a continuation method as described by, for example, [30].
(ii) Calculate the solution of (5.4), **u**_*s*_, using Newton’s method at *β* = *β*_*c*_.
(iii) Calculate the eigenfunction **g** and eigenvalue *s*_*c*_ = i*ω*_*c*_ using an eigensolver to solve **J**[**u**_*s*_]**g** = *s***M**[**u**_*s*_]**g**.
(iv) Calculate the Hessian products on the right-hand side of (5.11). If an exact expression for the Jacobian matrix is available, the Hessian products can be numerically computed using the central-difference formula
  5.18 $$H[u_{s}](v_{1},v_{2})\approx \frac{\text{J}[{\text{u}}_{s}+k_{1}{\text{v}}_{2}]{\text{v}}_{1}-\text{J}[{\text{u}}_{s}-k_{1}{\text{v}}_{2}]{\text{v}}_{1}}{2k_{1}},$$
  where *k*_1_ is a small finite difference parameter.
(v) Calculate the functions **φ**_0_, **φ**_1_ and **φ**_2_ in (5.11) using a linear solver.
(vi) Calculate the adjoint eigenfunction, $g^{†}$, by solving ${\text{J}}^{T}[{\text{u}}_{s}]{\text{g}}^{†}=-s{\text{M}}^{T}[{\text{u}}_{s}]{\text{g}}^{†}$.
(vii) Calculate the Hessian and third-order products in (5.16) using (5.18) by approximating the third-order operator (again assuming an exact Jacobian) as
  $$\begin{array}{rl} & T[u_{0}](v_{1},v_{2},v_{3})\\ & \;\approx \frac{\text{J}[{\text{u}}_{s}+k_{2}{\text{v}}_{2}+k_{2}{\text{v}}_{3}]{\text{v}}_{1}-\text{J}[{\text{u}}_{s}+k_{2}{\text{v}}_{2}]{\text{v}}_{1}-\text{J}[{\text{u}}_{s}-k_{2}{\text{v}}_{2}+{\text{v}}_{3}]{\text{v}}_{1}+\text{J}[{\text{u}}_{s}-k_{2}{\text{v}}_{2}]{\text{v}}_{1}}{2k_{2}^{2}}. \end{array}$$
(viii) Finally, combine the quantities in (5.16) to calculate the values of $\hat{\nu },\hat{\lambda }$ and $\hat{\mu }$ given in (5.15), defining the discrete inner product as 〈**f**, **g**〉 = **fg**^*T*^.

We now return our focus to the specific equations in (2.1). These are discretized by the finite-element method, using the open-source software oomph-lib [27], in which the functions required for the above procedure have all been implemented. It is convenient to choose the independent parameter *β* = *Q*^−1^ and, to ensure the finite difference formulae stated above converge, *k*_1_ = 10^−4^ and *k*_2_ = 10^−3^. When *V* = *πr*^2^, with *r* = 0.46, *α* = 40, *w* = 0.25 and *h* = 0.024, this procedure was implemented and the critical values of *Q*_*c*_, *ω*_*c*_ and the Landau coefficients were found to three significant figures to be

5.19*a* $$\text{H}1:Q_{c}=Q_{1}=0.0301,\;\omega_{c}=3.58,\;\lambda =\delta (5.63-10.7\text{i}),\;\mu =1280-1200\text{i}$$

and

5.19*b* $$\text{H}2:Q_{c}=Q_{2}=0.0896,\;\omega_{c}=10.8,\;\lambda =\delta (-5.43-15.8\text{i}),\;\mu =-6150-1320\text{i}.$$

We have checked that the quadrant in the complex plane in which *λ* and *μ* lie (setting *δ* constant) does not change as parameters are varied thus not affecting the criticality of the bifurcation.

At this point, we wish to analyse solutions to (5.17). We note that a decomposition into modulus argument form, i.e. $A=\hat{r}(t)\exp(\text{i}\theta )$ converts the equation into a pair of ODEs:

5.20 $$\frac{\text{d}\hat{r}}{\text{d}t_{1}}=a\hat{r}+b{\hat{r}}^{3}\;\text{and}\;\frac{\text{d}\theta }{\text{d}t_{1}}=c+d{\hat{r}}^{2},$$

where a=ℜ(λ), b=ℜ(μ), c=ℑ(λ) and d=ℑ(μ). The invariant solutions occur when $\text{d}\hat{r}/\text{d}t_{1}=0$. The solutions corresponding to $\hat{r}=0$ are the steady states of the fully nonlinear steady equations, while the solution corresponding to $\hat{r}=\sqrt{-a/b}$ is an invariant periodic orbit of the fully nonlinear system. By examining the signs of *a* and *b* at each Hopf point and performing a stability analysis on the equations in (5.20) it is straightforward to show that H1 is subcritical: UPOs exist when *Q* > *Q*_1_ for *Q* near to *Q*_1_. Similarly, H2 is supercritical so stable periodic orbits exist when *Q* > *Q*_2_.

Near the H1 and H2 points the steady-state solution of (5.1), corresponding to $\hat{r}=0$ in (5.20), can be written in terms of the state variables as

5.21 $$u=u_{s}+\varepsilon^{2}\varphi_{2}+O(\varepsilon^{4}).$$

**Figure 9.**
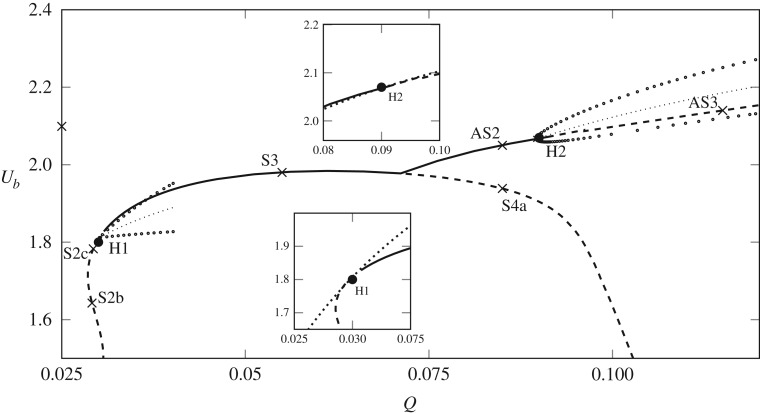
Comparison of the fully nonlinear solution space in the (*Q*, *U*_*b*_) plane with the weakly nonlinear approximation. The dotted lines indicate the centre of oscillation of the periodic orbits and the circular markers are the amplitude as given in (5.22). The solid and dashed curves in the inset diagrams (enlargements near H1 and H2) are the fully nonlinear steady solutions, while the black dotted curves are the weakly nonlinear approximations given by (5.21).

This weakly nonlinear approximation can be compared to the fully nonlinear steady-state bifurcation structure of §4. The dotted lines in the inset panels of figure 9 are the approximations given by (5.21). The approximation near the H2 bifurcation point is excellent in this projection; the fully nonlinear and weakly nonlinear curves are visually indistinguishable. For the H1 bifurcation point, the weakly nonlinear steady-state approximation deviates significantly away from the solution branch as we move away from H1 in the (*Q*, *U*_*b*_) projection. This difference is likely due to the existence of the fold, F2, in the immediate vicinity of H1. As *h* is varied, to approach the fold-Hopf point, a weakly nonlinear analysis should include the eigenmode associated with the zero eigenvalue and hence the form of the perturbation equation in (5.17) would differ.

The periodic orbits, corresponding to $\hat{r}=\sqrt{-a/b}$, can be stated as

5.22 $$u=u_{s}+\varepsilon \sqrt{-\frac{a}{b}}\;g{\text{e}}^{\text{i}(\omega_{c}t_{0}+\Delta )}+\varepsilon^{2}\left(\varphi_{2}-\frac{a}{b}\varphi_{1}-\frac{a}{b}\varphi_{0}{\text{e}}^{2\text{i}(\omega_{c}t_{0}+\Delta )}\right)+O(\varepsilon^{3}),$$

where *θ* = *θ*_0_ at *t* = 0 and Δ = *θ*_0_ + ε^2^(*c* − *ad*/*b*)*t*_0_ represents the $O(\varepsilon^{2})$ correction to the phase of the oscillations. The location of the centre of oscillations (ignoring time-dependent terms in (5.22)) of these periodic orbits are shown as dotted lines in the main panel of figure 9. The circular markers in this diagram indicate the amplitude of the periodic orbits. The initial phase *θ*_0_ will not alter the amplitude or period of the periodic orbit.

**Figure 10.**
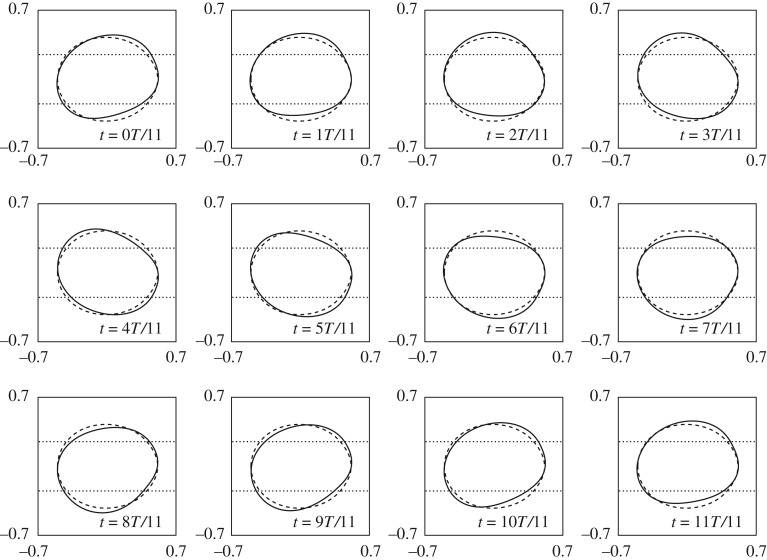
Unstable weakly periodic orbit near H1. The dashed shape is the fully nonlinear steady-state solution. Bubble shapes for $Q^{-1}=Q_{1}^{-1}(1-\varepsilon^{2})$, where *Q*_1_ is the critical value for H1 and ε = 0.2. The period is given by *T* = 2*π*/*ω* where *ω* = *ω*_*c*_ + ε^2^(*c* − *ad*/*b*), which in this case is *T* = 1.657.

An example of the weakly nonlinear bubble shapes of the periodic orbits near the H1 point is shown in figure 10. The oscillations are characterized by an oscillation about the symmetric steady solution and the oscillations are visible on both sides of the bubble. It can be seen that after half a period the bubble has ‘flipped’ from its initial shape. The weakly nonlinear equations in (5.20) predict that if the initial amplitude, *r*_0_, of a perturbation is smaller than a critical amplitude, $\hat{r}$, then the system will evolve towards *r* = 0, i.e. the S3 steady state. By contrast, if $r_{0}>\hat{r}$ then the trajectory will diverge to *r* → ∞. The H1 periodic orbit can, therefore, be considered an edge state of the reduced system as it forms the boundary between stable and unstable behaviour.

**Figure 11.**
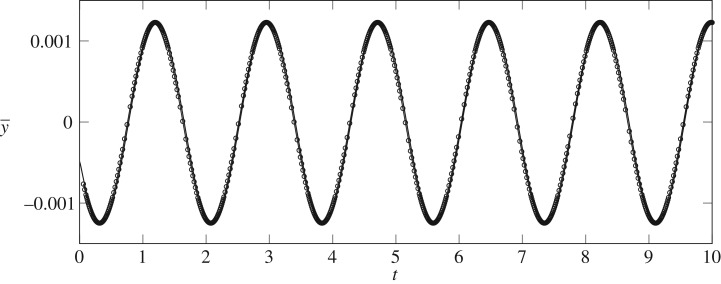
Comparison of the time signal of $\bar{y}$ between the fully nonlinear time-dependent solutions (solid lines) and the weakly nonlinear solutions (symbols) near H1. $Q^{-1}=Q_{1}^{-1}(1-\varepsilon^{2})$ with ε = 0.01.

**Figure 12.**
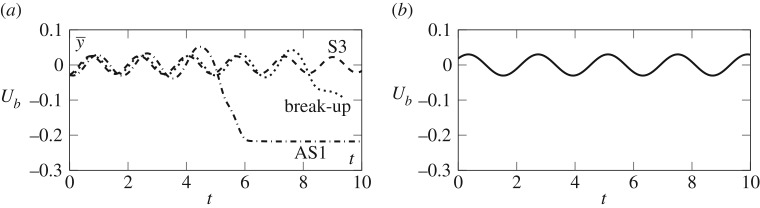
Comparison between the fully nonlinear time-dependent solutions and the weakly nonlinear solutions near H1, *Q* = 0.032. (*a*) The time signal of $\bar{y}$ for the fully nonlinear simulations. The dashed line is for *θ*_0_ = *π*/2. The dotted line is for *θ*_0_ = 5*π*/12 where the bubble eventually breaks up and the dashed-dotted line is for *θ*_0_ = *π*/4 when the bubble reaches the AS1 steady state. (*b*) The weakly nonlinear simulations when *θ*_0_ = 0.

The orbits near H1 are unstable so as noted in §4 we are unable to precisely capture the fully nonlinear periodic orbit by time-integration alone. However, by choosing the weakly nonlinear solution in (5.22) as an initial condition to the fully nonlinear time-dependent problem, a qualitative comparison can be made. A characteristic feature of the UPOs is that the perturbation oscillates all the way around the edge of the bubble (figure 10). These oscillations will cause oscillations in the centroid of the bubble. Therefore a useful way to compare the fully nonlinear simulations and weakly nonlinear approximations is to measure the time signal of $\bar{y}$ so that the amplitude and period can be compared. A result of one such simulation when ε = 0.01 is shown in figure 11 in the fully nonlinear simulations (solid lines) and weakly nonlinear simulations (circular symbols). The period and amplitude of oscillations of both regimes are nearly identical in both regimes. At this value of ε, we are very close to the H1 point and hence the oscillations in the fully nonlinear regime will take a long time to decay. For larger values of ε, we find that depending on the initial phase of the initial condition, the bubble will either break up, evolve to the AS1 solution or continue to oscillate towards the S3 solution (figure 12). This is further evidence that the H1 orbit is an edge state in the fully nonlinear regime. In contrast to the weakly nonlinear regime, there are three possible outcomes with the fully nonlinear H1 periodic edge state forming the dividing line between stable symmetric evolution and either break-up or attraction towards the stable asymmetric states.

**Figure 13.**
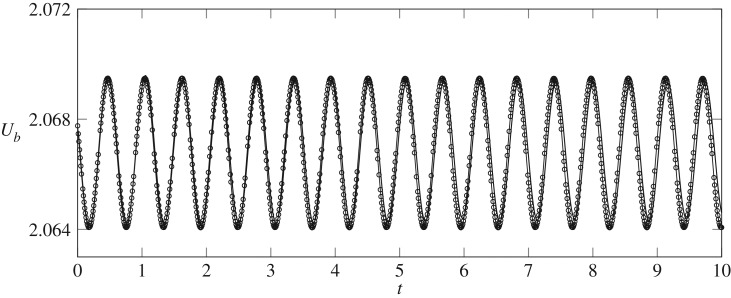
Comparison of the time signal of *U*_*b*_ between the fully nonlinear time-dependent solutions (solid line) and the weakly nonlinear solutions (symbols) near H2. $Q^{-1}=Q_{2}^{-1}(1-\varepsilon^{2})$ with ε = 0.01.

An example of the weakly nonlinear bubble shapes of the periodic orbits near the H2 point is shown in figure 6; in this case, the underlying steady state is asymmetric. The stable periodic orbit near the H2 point is characterized by oscillations appearing on the single side of the bubble that is over the edge of the depth-perturbation. These periodic orbits are linearly stable and hence time-integration of the fully nonlinear system should give a good indication of the period and amplitude of the orbit as *t* progresses. As the centroid of the bubble shape does not vary significantly, a more appropriate time signal is *U*_*b*_, as shown in figure 13. The period and amplitude of the weakly nonlinear periodic orbits demonstrate excellent agreement with the fully nonlinear time-dependent simulations.

## 6. Conclusion

We have investigated the transient dynamics and invariant solutions of a finite air bubble propagating in a perturbed Hele-Shaw channel, a model system in which to explore complex transition scenarios in fluid mechanics. In §3, time-dependent simulations confirmed that a number of different modes of propagation are possible, including multi-tipped solutions and oscillatory solutions as the flow rate increases. These simulations and an analysis of the steady solution space revealed a finite width region of bistability (with respect to steady states). The demarcation of this bistable region by two Hopf bifurcations explains the oscillatory solutions observed from the time simulations. The first Hopf bifurcation, H1, is subcritical, resulting in the emergence of UPOs and the second, H2, is supercritical, resulting in stable periodic orbits. An interesting feature is that the nature of the bistability changes as *Q* increases. Initially (*Q* immediately larger than *Q*_1_) a slower symmetric steady state, S3, coexists with an ever-present asymmetric steady state, AS1. A transition occurs at the pitchfork bifurcation as the stable AS2 asymmetric branch emerges. Finally, due to the supercritical Hopf bifurcation, H2, a stable periodic orbit appears as an alternative stable invariant solution to the asymmetric AS1 branch.

The criticality of the Hopf bifurcations was determined and periodic orbits were approximated using a weakly nonlinear stability analysis in §5. The method described here applies to a range of governing equations and is independent of the choice of discretization. It has been implemented in a general form in the open-source library oomph-lib. The approximate periodic orbits were in good agreement with the fully nonlinear transient simulations. The oscillations of the UPO near H1 manifest themselves as oscillations all the way around the bubble that cause an oscillatory change in the centroid, $\bar{y}$, of the bubble. For flow rates just above the H2 bifurcation point, stable periodic orbits exist. The oscillations now appear as ‘waves’ on the side of the bubble above the depth-perturbation only, with the lower edge of the bubble ‘resting’ on the lower limits of the depth-perturbation. In this case, the offset of the bubble centroid remains non-zero and largely constant and the underlying steady state is asymmetric. These stable, asymmetric oscillations have similar characteristics to the oscillations found for air-fingers by [4–6,8] and can be viewed as the finite-bubble analogue. The oscillations are driven by interactions between the edge of the bubble and the edge of the constriction, which is typically only possible for asymmetric bubble shapes. By contrast, the UPOs near H1 have not been seen before and appear to be a new phenomena in this system. In this case, the oscillations may be driven by underdamping in the mechanism that restores a symmetric bubble to the centre of the channel after perturbation; the generic mechanism requires an interaction between bubble deformation and surface tension and is described in [11]. In preliminary experiments, we have observed both persistent (stable) H2 orbits and transient (unstable) H1 orbits.

The periodic orbits near the H1 bifurcation point, although unstable, are important in understanding the full time-dependent behaviour of the system. Figure 12*a* provides evidence that these UPOs are edge states of the system (see, for example [12,43–45]). Slight changes in the initial condition can result in either the bubble returning to the steady S2 state or breaking up/evolving to the AS1 solution. The evidence presented here shows that the UPO is on the boundary between these two forms of transient behaviour. We have used a weakly nonlinear approximation to quantify UPOs and edge states rather than using edge-tracking techniques that have been applied to shear flows [12,43] and droplet pinch-off [16]. These edge-tracking techniques rely on there being two distinct types of transient behaviour. An added complication in the present case is that there are three possible outcomes: the bubble returning to the S2 steady state; the bubble breaking up; or the bubble evolving to the AS1 symmetric state. In a standard Hele-Shaw channel (*h* = 0), the steady solution structure is different so we would not expect the exact same transient behaviour. From a broader perspective, the importance of UPOs in influencing the dynamics of transitional fluid flow has been demonstrated in plane Couette flow [44] and in wall-bounded shear flows [45] via direct numerical simulation of the Navier–Stokes equations. More recently, Dynamic Mode Decomposition and Koopman analysis have been used to provide a method for identifying UPOs in high-dimensional systems [46,47]. The work presented here presents an alternative method for identifying UPOs in high-dimensional fluid flow systems and for understanding their significance in transitional phenomena.

We would expect to see evidence of the periodic orbits and the associated transitions in the bistable region in any experimental results. The UPO near H1 is therefore highly influential in the underlying dynamics of a bubble. Understanding the region of influence of the UPO and a full comparison of the sensitivity of bubble break-up to initial conditions, especially close to bifurcation points, is the subject of a combined experimental and theoretical study currently underway.

## Footnotes

1 © The Japan Society of Fluid Mechanics. Reproduced by permission of IOP Publishing. All rights reserved. doi:10.1088/1873-7005/aaa5cf.

## Data accessibility

The source code for our calculations (along with instructions) can be found at https://figshare.com/s/254feb5ce70a340b0d57.
